# Prognostic accuracy of triage tools for adults with suspected COVID-19 in a prehospital setting: an observational cohort study

**DOI:** 10.1136/emermed-2021-211934

**Published:** 2022-02-09

**Authors:** Carl Marincowitz, Laura Sutton, Tony Stone, Richard Pilbery, Richard Campbell, Benjamin Thomas, Janette Turner, Peter A Bath, Fiona Bell, Katie Biggs, Madina Hasan, Frank Hopfgartner, Suvodeep Mazumdar, Jennifer Petrie, Steve Goodacre

**Affiliations:** 1 Centre for Urgent and Emergency Care Research (CURE), Health Services Research School of Health and Related Research (ScHARR), The University of Sheffield, Sheffield, UK; 2 Clinical Trials Research Unit (CTRU), Health Services Research School of Health and Related Research (ScHARR), The University of Sheffield, Sheffield, UK; 3 Yorkshire Ambulance Service NHS Trust, Wakefield, UK; 4 Centre for Health Information Management Research (CHIMR) and Health Informatics Research Group, Information School, University of Sheffield, Sheffield, UK

**Keywords:** emergency care systems, COVID-19, triage, risk management, emergency ambulance systems

## Abstract

**Background:**

Tools proposed to triage patient acuity in COVID-19 infection have only been validated in hospital populations. We estimated the accuracy of five risk-stratification tools recommended to predict severe illness and compared accuracy to existing clinical decision making in a prehospital setting.

**Methods:**

An observational cohort study using linked ambulance service data for patients attended by Emergency Medical Service (EMS) crews in the Yorkshire and Humber region of England between 26 March 2020 and 25 June 2020 was conducted to assess performance of the Pandemic Respiratory Infection Emergency System Triage (PRIEST) tool, National Early Warning Score (NEWS2), WHO algorithm, CRB-65 and Pandemic Medical Early Warning Score (PMEWS) in patients with suspected COVID-19 infection. The primary outcome was death or need for organ support.

**Results:**

Of the 7549 patients in our cohort, 17.6% (95% CI 16.8% to 18.5%) experienced the primary outcome. The NEWS2 (National Early Warning Score, version 2), PMEWS, PRIEST tool and WHO algorithm identified patients at risk of adverse outcomes with a high sensitivity (>0.95) and specificity ranging from 0.3 (NEWS2) to 0.41 (PRIEST tool). The high sensitivity of NEWS2 and PMEWS was achieved by using lower thresholds than previously recommended. On index assessment, 65% of patients were transported to hospital and EMS decision to transfer patients achieved a sensitivity of 0.84 (95% CI 0.83 to 0.85) and specificity of 0.39 (95% CI 0.39 to 0.40).

**Conclusion:**

Use of NEWS2, PMEWS, PRIEST tool and WHO algorithm could improve sensitivity of EMS triage of patients with suspected COVID-19 infection. Use of the PRIEST tool would improve sensitivity of triage without increasing the number of patients conveyed to hospital.

**Key messages**

**What is already known on this subject**

- Current methods recommended to risk assess and determine if patients with suspected COVID-19 in the community and prehospital setting require treatment in hospital are consensus based.
- Triage tools such as the PRIEST score have been shown to accurately predict adverse outcomes in patients with suspected COVID-19 infection in the ED. Validation in a prehospital setting could aid clinical decision making.

**What this study adds**

- We retrospectively assessed the ability of five triage tools in patients evaluated in the prehospital setting by a single ambulance service. Any of the tools could potentially improve identification of patients with suspected COVID-19 infection who were at risk of adverse outcomes, compared with existing practice.
- NEWS2 (National Early Warning Score, version 2), PMEWS, PRIEST tool and WHO algorithm identified patients at risk of adverse outcome with high sensitivity (>0.95).
- Use of the PRIEST tool could lead to significant gains in sensitivity without increasing the number of patients conveyed to hospital.

## Background

Emergency Medical Service (EMS) and other urgent and emergency care practitioners assessing patients with suspected COVID-19 infection in the community must rapidly determine whether patients need treatment in hospital or can safely remain at home. The overall risk of mortality in patients with confirmed infection is around 1%, and if conveyance is too liberal, hospitals could be overwhelmed by patients who require no specific treatment.^1^ However, failing to identify a patient at risk of serious deterioration could lead to avoidable harm.^2^

Prognostic research has almost exclusively been conducted in hospital settings, and current national and international guidelines for risk stratification of patients with suspected COVID-19 in the community are consensus based.^1 3–5^ Clinical acuity scores, such as the UK Royal College of Physicians National Early Warning Score, version 2 (NEWS2), have been suggested in some guidelines as a way to risk stratify patients with suspected COVID-19 infection in the community.^6^ The WHO decision-making algorithm for respiratory infection and CRB-65 are used to risk stratify patients with bacterial pneumonia and PMEWS for use in patients with influenza.^7–9^ However, the accuracy of these risk-stratification tools has only been validated in hospitalised or non-COVID populations.

NEWS2 has shown good prediction of adverse outcome in patients attending the ED with suspected COVID-19.^7^ The PRIEST tool was derived by adding age, sex and performance status to NEWS2, and validation showed improved prediction compared with NEWS2 alone.^10 11^ Validation of the PRIEST tool, NEWS2 and other clinical risk-stratification tools recommended for use in hospital in a community setting^7 10 12–14^ could identify the most accurate means to triage need for hospitalisation, thereby reducing unnecessary hospital attendances and improve the identification of those most at risk of serious adverse outcomes.

Our study aimed to:

1. Estimate the accuracy of risk-stratification tools recommended to predict severe illness in adults with suspected COVID-19 infection in a prehospital setting.
2. Compare the accuracy of risk-stratification tools to existing clinical decision making around transport to hospital.

## Methods

### Study design

This observational cohort study used linked routinely collected EMS data to assess the accuracy in a community setting of five clinical risk-stratification tools (PRIEST tool, NEWS2, WHO algorithm, CRB-65 and PMEWS) recommended for use in hospitalised patients with COVID-19 or similar respiratory infections (triage tools shown in online supplemental material 1).^7 10 12–14^

10.1136/emermed-2021-211934.supp1 Supplementary data

### Setting

Patients with suspected COVID-19 infection attended by EMS provided by Yorkshire Ambulance Service NHS Trust (YAS). EMS provided by YAS covers a region in the north of England of approximately 6000 square miles and with a population of 5.5 million.

### Data sources and linkage

EMS providers complete an electronic patient report form (ePRF) each time they attend an emergency call, which records presenting patient characteristics and clinical care in a standardised manner. YAS provided a dataset of ePRF data for all EMS responses between the 26 March 2020 and 25 June 2020 where the attending ambulance staff recorded a clinical impression of suspected or confirmed COVID-19 infection. The dataset consisted of patient identifiers, demographic data, measured physiological parameters, other available clinical information and the outcome of the assessment (including whether the patient was conveyed to hospital). Ambulance attendances were linked to routinely collected limited COVID-related general practice (GP) records, ED attendances, hospital inpatient admissions, critical care periods and death registrations from the UK Office of National Statistics (online supplemental material 2).

### Inclusion criteria

Our final cohort consisted of all adult (aged 16 years and over) patients at the time of first (index) EMS attendance between 26 March and 25 June 2020, in which the attending ambulance staff recorded a clinical impression of suspected or confirmed COVID-19 infection and who were successfully traced by NHS Digital.

### Outcome

The primary outcome was death, renal, respiratory or cardiovascular organ support (identified from death registration and critical care data) at 30 days from index attendance. This includes basic and advanced cardiovascular and respiratory organ support such as high-flow oxygen administration, non-invasive ventilation and close monitoring due to the risk of respiratory failure in a higher dependency setting.

The secondary outcome was death up to 30 days from index contact.

### Patient characteristics

Physiological parameters were extracted from the first (primary) set of clinical observations recorded by the ambulance crew. Consistent with methods used to estimate the Charlson Comorbidity Index from the available routine data, comorbidities were included if recorded 12 months before the index EMS attendance.^15 16^ In a similar way, only immunosuppressant drug prescriptions documented in GP records within 30 days before the index attendance contributed to the immunosuppression comorbidity variable. Pregnancy status was based on GP records recorded in the previous 9 months. Frailty in patients older than 65 years was derived from the latest recorded Clinical Frailty Scale (CFS) score (if recorded) in the electronic GP records prior to index attendance.^17^ Patients under the age of 65 years were not given a CFS score since it is not validated in this age group. However, if a CFS score was required to calculate a triage tool and the patient was under the age of 65 years, it was assumed to be 1. Performance status was estimated from the CFS.

### Analysis

We retrospectively applied the five triage tools to our cohort to assess their accuracy for the primary and secondary outcomes.^7 10 12–14^ Online supplemental material 1 provides details of scoring and handling missing data for the triage tools. For each tool we plotted the receiver operating characteristic (ROC) curve and calculated the area under the ROC curve (c-statistic) for discriminating between patients with and without adverse outcomes. We calculated sensitivity, specificity, positive predictive value (PPV) and negative predictive value (NPV) at the following prespecified decision-making thresholds based on recommended or usual use: 0 vs 1+ CRB-65; 0–1 vs 2+ NEWS2; 0–2 vs 3+ PMEWS; 0–4 vs 5+ PRIEST; 0 vs 1 WHO score. A score of ≥1 for CRB-65 and the WHO score are recommended thresholds for indicating consideration of hospital admission in bacterial pneumonia.^18 19^ The NEWS2 and PMEWS thresholds used are lower than previously proposed (0–3 vs 4+ NEWS and 0–3 vs 4+ PMEWS) for triaging patient acuity and are based on the assessment of their performance in a UK ED population of patients with suspected COVID-19 infection, where higher thresholds gave suboptimal sensitivity.^11^ The threshold for the PRIEST score is also based on performance in this ED cohort.^11^ These tools were compared with the sensitivity, specificity, PPV and NPV of EMS clinicians’ decision to transfer patients to hospital. The International Severe Acute Respiratory Infection Consortium (Coronavirus Clinical Characterisation Consortium) prediction model was not validated as it requires investigations that are available in a hospital setting, including blood tests, and is intended for prediction of inpatient mortality.^20^ All analyses were based on assessment during the index EMS attendance and completed with SAS V.9.4.

### Sample size

We a priori assessed the required sample size on the estimated precision of the area under the ROC curve based on a likely 5% event rate in a cohort of 6000 patients (online supplemental material 3).^21^

### Patient and public involvement

The Sheffield Emergency Care Forum (SECF) is a public representative group interested in emergency care research.^22^ Members of SECF advised on the development of the PRIEST study and two members joined the Study Steering Committee. A PRIEST study patient-public involvement (PPI) group was created during the study which included patients who had been admitted to hospital with COVID-19 or their family members. Although not involved in data linkage or conducting the analyses, both PPI groups were consulted regarding study design, particularly the ethical implications of using routine health data for research. All study findings were presented and discussed with the PPI groups. Members helped with interpretation of findings particularly regarding acceptable risk of misclassification.

## Results

All totals presented from NHS Digital-derived datasets (sex, number of current medications, comorbidities, CFS and outcomes) are rounded to the nearest 5, with small numbers suppressed to comply with NHS Digital data disclosure guidance.

### Study population

**Figure 1:**
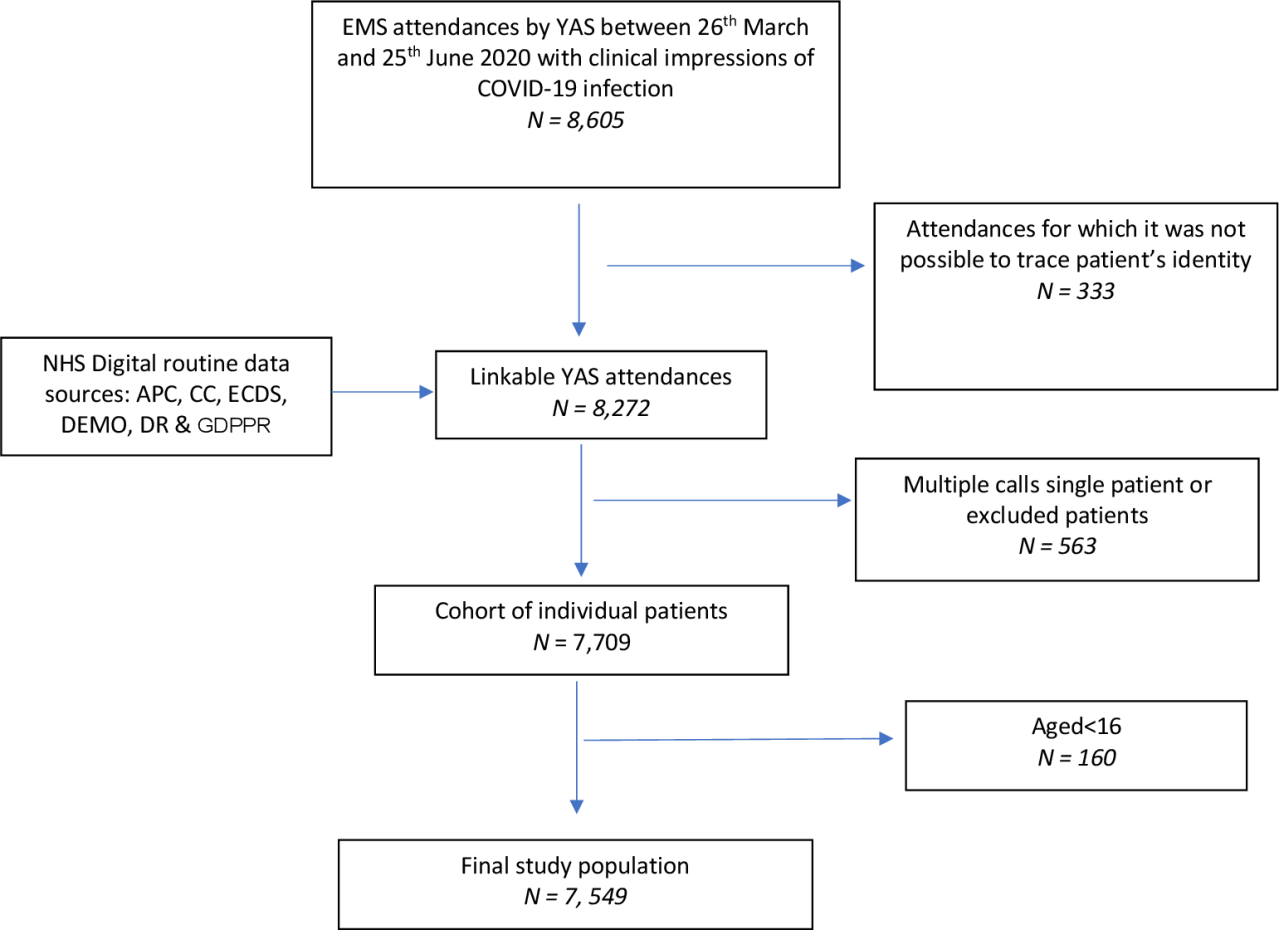
STROBE flow diagram of study population selection. EMS, Emergency Medical Service; STROBE, Strengthening the Reporting of Observational Studies in Epidemiology; YAS, Yorkshire Ambulance Service NHS Trust; APC, Admitted Patient Care; CC, Critical Care; ECDS, Emergency Care Data Set; DEMO, Demographics; DR, Death Registrations; GDPPR, General Practice Data for Pandemic Planning and Research.

Figure 1 and table 1 summarise study cohort derivation and the characteristics of 7549 included individual adult patients. In total, 1330 patients (17.6%, 95% CI 16.8% to 18.5%) experienced the primary outcome (death or organ support) and 1065 (14.1%, 95% CI 13.4% to 14.9%) the secondary outcome (death). Of the 7549 patients, the decision was made to transport 4905 (65%) to hospital at index attendance. Of those, 1120 (22.9%) experienced the primary adverse outcome. Of those not transported to hospital, 210 (7.9%) had an adverse outcome. Within the cohort, 3925 patients (52%, 95% CI 50.9% to 53.1%) were admitted as inpatients and 2785 (36.9%, 95% CI 35.8% to 38%) had a diagnosis of COVID-19 confirmed in hospital (since unrestricted community testing was not available until 18 May 2020) within 30 days of index EMS attendance.

**Table 1:** Patient characteristics by outcome

Characteristic	Statistic/level	Adverse outcome	No adverse outcome	Total
	N	1330 (17.6%)*	6220 (82.4%)*	7549
Age (years)*	Mean (SD)	74.5 (15.4)	56.9 (19.4)	60 (20)
	Median (IQR)	78 (65–86)	56 (42–73)	59 (45–77)
	Range	19–103	16–105	16–105
Sex*	Male	760 (57.3%)	2825 (45.4%)	3590 (47.5%)
	Female	570 (42.7%)	3390 (54.6%)	3960 (52.5%)
No of current medications*	N	1330	6220	7549
	Mean (SD)	4.5 (3.3)	3.2 (3.3)	3.4 (3.3)
	Median (IQR)	4 (2–7)	2 (0–5)	3 (0–6)
	Range	0–19	0–19	0–19
Comorbidities*	Cardiovascular disease	95 (7%)	290 (4.6%)	380 (5.1%)
	Chronic respiratory disease	375 (28%)	1855 (29.8%)	2230 (29.5%)
	Diabetes	390 (29.2%)	995 (16%)	1380 (18.3%)
	Hypertension	610 (45.8%)	1765 (28.4%)	2375 (31.4%)
	Immunosuppression	280 (21.1%)	930 (15%)	1215 (16.1%)
	Active malignancy	60 (4.6%)	115 (1.9%)	180 (2.3%)
	Renal impairment	55 (4.1%)	125 (2%)	180 (2.4%)
	Stroke	30 (2.3%)	85 (1.4%)	115 (1.5%)
Clinical frailty*	Not Applicable (age <65 years)	330 (47.5%)	3985 (86.4%)	4310 (81.3%)
	Missing	645	1605	2250
	1–3	20 (4.7%)	40 (6.4%)	60 (5.8%)
	4–6	75 (20.5%)	240 (37.7%)	310 (31.4%)
	7–9	270 (74.8%)	350 (55.9%)	620 (62.8%)
AVPU**	Missing†	13	58	71
	Alert	1002 (76%)	5860 (95.1%)	6862 (91.8%)
	Confusion	125 (9.5%)	188 (3.1%)	313 (4.2%)
	Voice	100 (7.6%)	84 (1.4%)	184 (2.5%)
	Pain	64 (4.9%)	21 (0.3%)	85 (1.1%)
	Unresponsive	27 (2%)	7 (0.1%)	34 (0.5%)
GCS	N	1297	6085	7382
	Mean (SD)	13.7 (2.4)	14.8 (0.8)	14.6 (1.3)
	Median (IQR)	15 (14–15)	15 (15–15)	15 (15–15)
	Range	3–15	3–15	3–15
Diastolic BP (mm Hg)	N	1278	6029	7307
	Mean (SD)	76.7 (17.7)	84.5 (15.9)	83.1 (16.5)
	Median (IQR)	76 (65–87)	84 (74–94)	83 (72–93)
	Range	0–193	22–167	0–193
Systolic BP (mm Hg)	N	1277	6032	7309
	Mean (SD)	133.2 (25.8)	140.2 (23.2)	139 (23.9)
	Median (IQR)	132 (116–148)	139 (124–153)	138 (123–152)
	Range	65–238	33–237	33–238
Pulse rate (beats/min)	N	1303	6130	7433
	Mean (SD)	100.2 (22.5)	96 (19.5)	96.7 (20.1)
	Median (IQR)	99 (84–115)	94 (82–109)	95 (82–110)
	Range	38–194	7–190	7–194
Respiratory rate (breaths/min)	N	1315	6145	7460
	Mean (SD)	30.1 (10)	23.1 (6.9)	24.4 (8)
	Median (IQR)	28 (22–36)	20 (18–26)	22 (18–28)
	Range	0–76	0–84	0–84
Oxygen saturation	Missing	36	109	145
	>95% on air	142 (11%)	3532 (57.8%)	3674 (49.6%)
	94%–95% on air	134 (10.3%)	854 (14%)	988 (13.3%)
	92%–93% on air	109 (8.4%)	449 (7.3%)	558 (7.5%)
	<92% on air or O_2_ given	910 (70.3%)	1274 (20.9%)	2184 (29.5%)
Blood glucose (mmol/L)	N	982	4021	5003
	Mean (SD)	8.1 (4)	6.9 (3.2)	7.2 (3.4)
	Median (IQR)	6.8 (5.6–9)	6 (5.2–7.3)	6.2 (5.2–7.7)
	Range	0.9–35	1.1–33.8	0.9–35
Temperature (°C)	N	1301	6115	7416
	Mean (SD)	38.1 (1.2)	37.8 (1.1)	37.8 (1.1)
	Median (IQR)	38.2 (37.4–38.9)	37.7 (37–38.5)	37.8 (37–38.6)
	Range	32–42	34–41.7	32–42

*To comply with NHS Digital disclosure guidance, totals for these variables are rounded to the nearest 5 which may result in apparent disparities in the overall totals.

†** Alert, Voice, Pain,

### Triage tool performance

**Figure 2:**
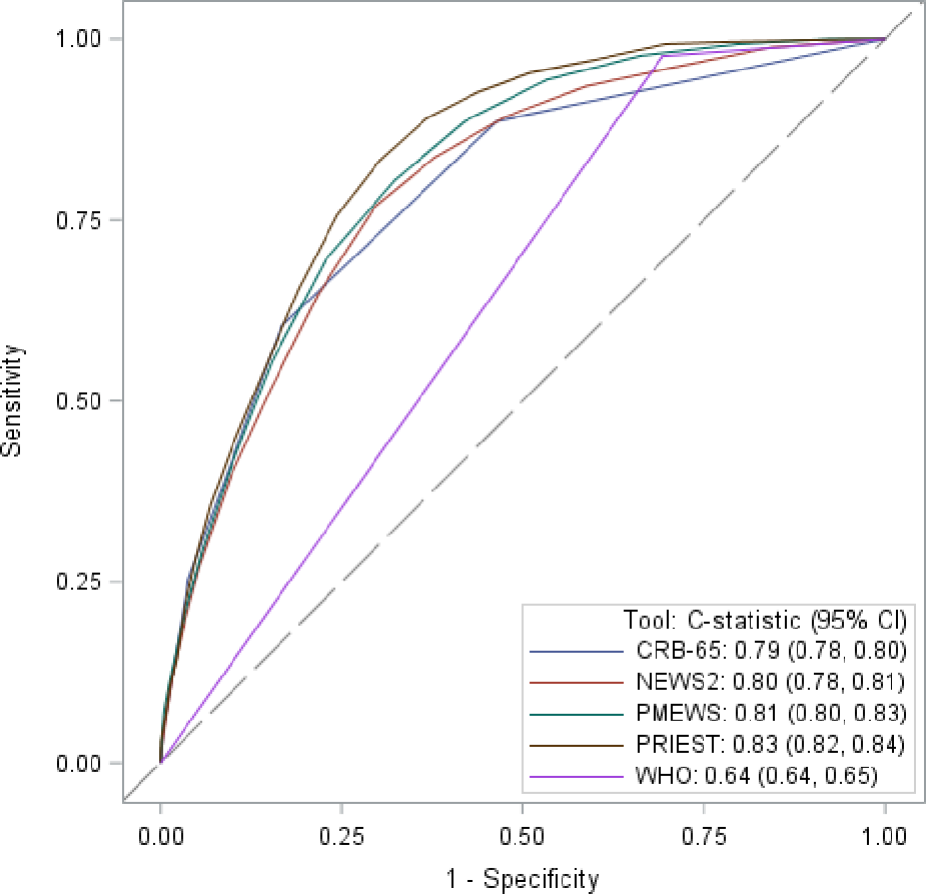
Receiver operating characteristic curves showing triage tool performance for predicting any adverse outcome.

**Figure 3:**
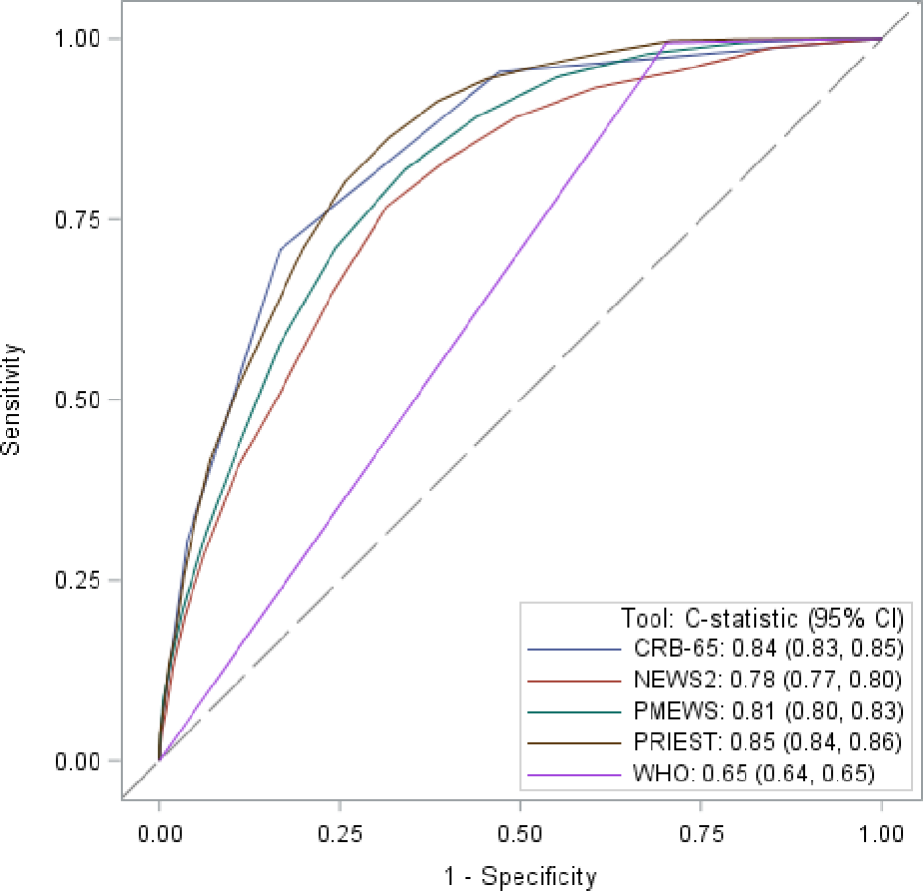
Receiver operating characteristic curves showing triage tool performance for predicting death within 30 days.

Sensitivity, specificity, PPV and NPV for predicting the primary composite outcome using predefined score thresholds are provided in table 2, and the secondary outcome of death is given in table 3. Sensitivity and specificity statistics are provided for every score threshold in online supplemental material 4, and calibration plots for the primary outcome in online supplemental material 5 (WHO algorithm excluded as the binary nature of the decision rule prevented calculation). The ROC curves for these analyses are shown in figures 2–3).

**Table 2:** Triage tool diagnostic accuracy statistics (95% CI) for predicting any adverse outcome

Tool	N*	n (%) adverse outcome†	C-statistic	Threshold	n (%) above threshold	Sensitivity	Specificity	PPV	NPV
CRB-65	7469	1315 (18)	0.79 (0.78 to 0.80)	>0	4010 (54)	0.89 (0.88 to 0.89)	0.54 (0.53 to 0.54)	0.29 (0.29 to 0.30)	0.96 (0.95 to 0.96)
NEWS2	7433	1315 (18)	0.80 (0.78 to 0.81)	>1	5574 (75)	0.96 (0.96 to 0.96)	0.30 (0.29 to 0.30)	0.23 (0.22 to 0.23)	0.97 (0.97 to 0.97)
PMEWS	7460	1315 (18)	0.81 (0.80 to 0.83)	>2	5352 (72)	0.98 (0.97 to 0.98)	0.34 (0.33 to 0.34)	0.24 (0.24 to 0.24)	0.99 (0.98 to 0.99)
PRIEST	7471	1315 (18)	0.83 (0.82 to 0.84)	>4	4932 (66)	0.97 (0.97 to 0.97)	0.41 (0.40 to 0.41)	0.26 (0.25 to 0.26)	0.98 (0.98 to 0.99)
WHO	7471	1315 (18)	0.64 (0.64 to 0.65)	>0	5539 (74)	0.98 (0.97 to 0.98)	0.31 (0.30 to 0.31)	0.23 (0.23 to 0.24)	0.98 (0.98 to 0.99)

*Patients with less than three parameters were excluded from analysis when estimating performance.

†Totals rounded to nearest 5.

NEWS2, National Early Warning Score, version 2; NPV, negative predictive value; PPV, positive predictive value.

**Table 3:** Triage tool diagnostic accuracy statistics (95% CI) for predicting death within 30 days

Tool	N*	n (%) 30 days of death†	C-statistic	Threshold	n (%) above threshold	Sensitivity	Specificity	PPV	NPV
CRB-65	7469	1055 (14)	0.84 (0.83 to 0.85)	>0	4010 (54)	0.95 (0.95 to 0.96)	0.53 (0.53 to 0.54)	0.25 (0.25 to 0.26)	0.99 (0.98 to 0.99)
NEWS2	7433	1055 (14)	0.78 (0.77 to 0.80)	>1	5574 (75)	0.95 (0.95 to 0.96)	0.28 (0.28 to 0.29)	0.18 (0.18 to 0.18)	0.97 (0.97 to 0.98)
PMEWS	7460	1050 (14)	0.81 (0.80 to 0.83)	>2	5352 (72)	0.98 (0.98 to 0.98)	0.32 (0.32 to 0.33)	0.19 (0.19 to 0.20)	0.99 (0.99 to 0.99)
PRIEST	7471	1055 (14)	0.85 (0.84 to 0.86)	>4	4932 (66)	0.98 (0.97 to 0.98)	0.39 (0.39 to 0.40)	0.21 (0.20 to 0.21)	0.99 (0.99 to 0.99)
WHO	7471	1055 (14)	0.65 (0.64 to 0.65)	>0	5539 (74)	0.99 (0.99 to 1.00)	0.30 (0.30 to 0.30)	0.19 (0.19 to 0.19)	1.00 (1.00 to 1.00)

*Patients with less than three parameters were excluded from analysis when estimating performance.

†Totals rounded to nearest 5.

NEWS2, National Early Warning Score, version 2; NPV, negative predictive value; PPV, positive predictive value.

EMS decision to transfer patients to hospital had a sensitivity of 0.84 (95% CI 0.83 to 0.85) and specificity 0.39 (95% CI 0.39 to 0.40) for the primary outcome. PPV was 0.23 (95% CI 0.22 to 0.23) and NPV was 0.92 (95% CI 0.92 to 0.92). Hypothetical use of any of the five triage tools would have achieved a higher sensitivity than the decision to transfer to hospital by the EMS crews within the cohort, but in the case of NEWS2, WHO algorithm and PMEWS, this was at a cost of a lower specificity (table 2). Of the tools assessed at the predetermined thresholds, CRB-65 achieved the highest specificity but at the cost of sensitivity, and the PRIEST tool achieved a balance between sensitivity, specificity and C-statistic of 0.83 (95% CI 0.82 to 0.84). The triage tools generally demonstrated better discrimination (except NEWS2) and a higher sensitivity for the secondary outcome, but a lower specificity (table 3). However, when predicting inpatient admission, the tools showed lower sensitivity, higher specificity and similar overall discrimination (online supplemental material 6). A lower threshold could be used for the PRIEST score, NEWS2 and PMEWS to increase the sensitivity, at the cost of reduced specificity, for this less critical outcome.

## Discussion

### Summary

The NEWS2, PMEWS, PRIEST tool and WHO algorithm identified patients at risk of adverse outcome with high sensitivity (>0.95) and specificity ranging between 0.3 (NEWS2) and 0.41 (PRIEST tool). They are, therefore, potentially suitable for use as triage tools to select patients for transfer to hospital. The high sensitivity of NEWS2 and PMEWS was achieved by using lower thresholds (NEWS2 0–1 vs 2+ and PMEWS 0–2 vs 3+) than previously recommended, based on performance in an ED population of patients with suspected COVID-19 infection.^11^

At index attendance, 65% of patients were transported to hospital. Although a useful comparator for triage tool performance, the observed accuracy of EMS decision making to transfer patients to hospital does not account for clinical best interest decisions not to covey patients to hospital who subsequently deteriorated, or patient wishes not to be conveyed. The sensitivity (0.84, 95% CI 0.83 to 0.85) and specificity (0.39, 95% CI 0.39 to 0.40) achieved by EMS decision making is nonetheless similar to that of tools used to triage undifferentiated patient acuity in the ED.^23^

To be clinically useful to EMS crews, the use of a triage tool would need to improve on the existing sensitivity of clinical decision making, thereby reducing the risk of not transporting a patient to hospital who subsequently deteriorates, without leading to a disproportionately large increase in hospital conveyance. Use of any of the five triage tools at the prespecified thresholds would potentially improve on the sensitivity of existing EMS decision making. However, the use of PMEWS, WHO algorithm or NEWS2 would lead to up to a 10% increase in ED conveyances (table 2). Use of both CRB-65 and the PRIEST score would lead to improvements in sensitivity without sacrificing specificity. CRB-65 achieved the highest specificity of any of the tools (0.54, 95% CI 0.53 to 0.54), and its use would reduce the number of patients conveyed to hospital by around 10%. However, patients not conveyed to hospital would have around a 4% risk of subsequently deteriorating. The PRIEST tool achieved a sensitivity of 0.97 (95% CI 0.97 to 0.97) without increasing the number of patients transported to hospital. Using the PRIEST tool, patients who were not conveyed to hospital would have a 2% risk of subsequent deterioration (compared with an estimated 8% on EMS decision making in this cohort).

### Strengths and limitations

Previous evaluations of triage tool accuracy and prognostic COVID-19 prognostic research in the prehospital setting are limited by only including patients who were subsequently admitted to hospital.^24–27^ This is the first evaluation to use a large cohort of patients identified from routinely collected EMS records and linked to nationally collected, patient-level, healthcare data to provide robust outcome data for all patients including those not conveyed to hospital. We had low rates of missing data in the variables used in the triage tools assessed (table 1). The PRIEST tool was robust to the removal of the performance status parameter; when doing so the C-statistic remained at 0.83 (95% CI 0.82 to 0.84). We also assessed the performance of triage tools in a cohort of patients with suspected infection which, in the absence of accurate universally available rapid COVID-19 diagnostic tests, reflects the population which EMS staff must clinically triage. Most existing research either aimed to determine if patients with suspected infection have COVID-19 or to risk stratify patients with confirmed infection in a hospital setting.^28^

Our evaluation of triage tool accuracy is limited to a single ambulance service, although one covering a large population across the North of England, so the results may not be generalisable to other healthcare settings. Other ambulance services may serve populations with a different risk profile, provide different types of EMS response or have different thresholds and guidelines regarding when to convey patients to hospital. The population used is likely to have similar baseline characteristics to that used to derive and validate the PRIEST score in an ED population, as it was conducted at the same time at hospitals in the region (and elsewhere in the UK).^10^ A sensitivity analysis in which patients recruited to the ED-based PRIEST study were removed from analysis, did not affect estimates of triage tool performance. The PRIEST tool may perform less well if applied to a different, especially non-UK, healthcare setting.

We assumed that if comorbidities were not recorded in routine data within the previous 12 months of the index event, then they were not present. Our cohort is based on the clinical impression of likely COVID-19 infection as determined by EMS crews. This is partly determined by the prevalence of COVID-19 infection which varied during the study period; however, YAS guidance stated possible COVID-19 infection should be considered in all patients with shortness of breath, cough or fever and in patients with a history of close contact with someone with these symptoms.

Our analysis is conducted on a cohort of patients that predates COVID-19 vaccination and improved community testing in the UK. This may affect the population characteristics of those with suspected COVID-19 being assessed by EMS crews. Older adults with COVID-19 may now have a lower risk of serious adverse outcomes due to vaccination than when our data were collected (over 90% of adults aged 70 years or above are now vaccinated in the UK), while infections are now more common in younger unvaccinated adults.^29^ This could reduce the prevalence of the primary study outcome. This would not necessarily affect the estimated sensitivity and specificity of the assessed triage tools but could affect the reported NPV and PPV. Increased community testing could reduce the proportion of patients with symptoms from respiratory diseases other than COVID-19 suspected of having COVID-19 when assessed by EMS crews. In the ED cohort of the PRIEST study, patients with COVID-19 had approximately double the mortality rate and rate of organ support compared with patients with other respiratory illnesses.^30^ We retrospectively applied risk-stratification tools to our cohort and they may perform differently if used by EMS crews. Further prospective validation may be required to assess the impact of implementation and determine the optimal threshold for clinical use of triage tools to account for changes in the population of patients with suspected COVID-19 infection.

### Implications

Clinical tools should be used in conjunction with clinical decision making when determining whether a patient needs to be conveyed to hospital by EMS crews. As previously highlighted, there may be good clinical reasons why patients who subsequently deteriorated were not conveyed to hospital in this cohort. It may also be appropriate based on initial EMS assessment, to leave a patient at home on first attendance, who then deteriorates. Within these limitations which may give the tested triage tools an apparent advantage over observed EMS practice, our study provides evidence that the use of existing clinical triage tools may improve clinical decision making in a prehospital setting where the prevalence of serious adverse outcomes is similar to the ED.

In healthcare contexts where minimising risk of adverse outcomes in those not conveyed to hospital is the priority, the use of PMEWS or the WHO criteria may be recommended, as they appear to optimise sensitivity. Use of the COVID-specific PRIEST tool would achieve almost the same gains in sensitivity (0.97 vs 0.98) without leading to a corresponding increase in patients being unnecessarily conveyed to hospital. The use of CRB-65 would maximise specificity over gains in sensitivity, with a 4% risk of adverse outcomes in patients left to self-care in the community. This may be appropriate in resource-constrained healthcare contexts, and as oxygen saturations do not form part of the assessment tool, it can be practically applied to a large range of healthcare settings.

Further research assessing triage tool performance alongside clinical judgement in the prehospital setting would be helpful to determine whether triage tools would improve accuracy of decisions to transfer patients to hospital in practice. In November 2020, the PRIEST tool was used within the YAS senior clinical support cell to assist with clinical decision making when providing advice to EMS crews. Support cell advice was based on the risk of subsequent deterioration (as estimated by the PRIEST score) alongside other patient factors including patient preference and best interest decision making at the end of life. Given the high prevalence of adverse outcomes in this cohort, the findings may not be applicable to other lower risk community settings (eg, patients being assessed by general practitioners) and therefore similar research is needed for these populations.

## Conclusion

The NEWS2, PMEWS, PRIEST tool and WHO algorithm achieved high estimated sensitivities with respect to death or organ support. Although there may be good clinical reasons why patients who deteriorated were not conveyed to hospital, the use of any these triage tools would potentially reduce the risk of false-negative triage and non-conveyance of a patient who subsequently deteriorates. Use of NEWS2, PMEWS and WHO algorithm would increase the proportion of conveyed patients, while the PRIEST tool could lead to significant gains in sensitivity without increasing the number of patients conveyed to hospital.

## Data Availability

Data may be obtained from a third party and are not publicly available. The data used for this study are subject to data sharing agreements with NHS Digital and YAS which prohibits further sharing of individual level data. The datasets used are obtainable from these organisations subject to necessary authorisations and approvals.
