# Supplementary material for: Prognostic accuracy of triage tools for adults with suspected COVID-19 in a prehospital setting: an observational cohort study
Source: Emerg Med J. 2022 Feb 8:emermed-2021-211934. doi: 10.1136/emermed-2021-211934 (PMC8844966; doi:10.1136/emermed-2021-211934)
Supplement: Supplementary file 1 [file emermed-2021-211934supp001.pdf]

Supplementary Material 1: Triage tool scoring details

CRB-65:

The CRB-65 score uses four parameters, each scoring 1 point when positive and zero if negative, to give a total score between zero and five.

Four parameters:

1. Confusion: GCS-V is less than 4 or GCS total is less than 15 or AVPU is recorded as V, P or U
2. Respiratory: rate of 30 breaths per minute or more
3. Blood pressure: diastolic BP is 60mmHg or less or systolic BP is 90 mmHg or less
4. Age: 65 years or more

Missing data: The rules above effectively classify missing data as normal. The CRB-65 score is recommended for community settings where access to blood testing is more limited. It is a 4-point scale that does not include urea with the threshold as <2 for low risk, 2+ for high risk. If a patient has fewer than three of the five parameters complete, the score was not be calculated.

PMEWS:

PMEWS uses six physiological parameters and patient parameters to calculate a score from zero to 19. The score is calculated by taking the score in the table below dependent on each of the six physiological parameters then adding points for two patient parameters after if they are positive.

Physiological:

**Score**	**3**	**2**	**1**	**0**	**1**	**2**	**3**
**Respiratory Rate**	≤8			9-18	19-25	26-29	≥30
**SaO_2**	<89	90-93	94-96	>96			
**Pulse Rate**	≤40	41-50		51-100	101-110	111-129	≥130
**Systolic BP**	≤70	71-90	90-100	>100			
**Temperature**		≤35.0	35.1-36.0	36.1-37.9	38-38.9	≥39	
**Neuro**				Alert	Confused Agitated*	Voice	Pain Uncon

* confused/agitated will be defined based on GCS-V<4 or GCS total<15

Patient:

1. Add 1 point if age>65

2. Add 1 point if either:
    a. Patient lives alone / no fixed abode or
    b. has a co-morbidity (respiratory, cardiac, renal, immunosuppressed, diabetes)
    c. performance status is more than two suggesting limited activity can self-care, limited activity limited self-care, or bed/chair bound no self-care.

Missing data: If data is missing one or two variables then the normal score (zero) was assumed. If more than three variables are missing the patient was excluded. These rules effectively classify missing data as normal. If AVPU was missing and GCS was recorded, impute the following AVPU scores using GCS: 0 if GCS=15, 1 if GCS=12-14; 2 if GCS=9-11 and 3 if GCS<9.

NEWS2:

The NEWS2 has seven parameters which are scores from zero to three providing an overall score between zero and 20. The scores for each parameter can be found in the table below.

Score	3	2	1	0	1	2	3
**Respiratory Rate**	≤8		9-11	12-20		21-24	≥25
**SaO_2**	≤91	92-93	94-95	≥96			
**Pulse Rate**	≤40		41-50	51-90	91-110	111-130	≥131
**Systolic BP**	≤90	91-100	101-110	111-219			≥220
**Temperature**	≤35.0		35.1-36.0	36.1-38.0	38.1-39.0	≥39.1	
**Neuro**				Alert			Confusion, Voice, Pain, Unresponsive
**Air or Oxygen**		Oxygen (based on FiO_2>21%, or FiO_2>0 L/min)		Air			

Missing data: Any missing data will be imputed with the value zero, therefore classifying missing as normal. The score was calculated if fewer than three of the parameters were available.

WHO decision making algorithm for hospitalisation with pneumonia:

The WHO decision making algorithm for hospitalisation with pneumonia suggests an adult patient is admitted (score 1) if any of the following are present:

- respiratory rate >30/minute,
- oxygen saturation <90%,
- respiratory distress (not included in this evaluation),
- age >60,
- any of the following comorbidities; hypertension, diabetes, cardiovascular disease, chronic respiratory disease, renal impairment or immunosuppression

As a subjective clinical assessment of respiratory distress is not routinely consistently recorded in our data this was not be included. Any missing data was assumed as normal. A score was not calculated if fewer than three of the above were complete.

The PRIEST clinical severity Score:

The core PRIEST clinical severity score consists of the seven parameters of NEWS2 and age, sex and performance status. We will assessed the full score and a version which omits performances status as this may not be reliably available from GP records. Scores for each parameter are as follows.

**Variable**	**Range**	**Score**
Respiratory rate (per minute)	12-20	0
	9-11	1
	21-24	2
	<9 or >24	3
Oxygen saturation (%)	>95	0
	94-95	1
	92-93	2
	<92	3
Heart rate (per minute)	51-90	0
	41-50 or 91-110	1
	111-130	2
	<41 or >130	3
Systolic BP (mmHg)	111-219	0
	101-110	1
	91-100	2
	<91 or >219	3

Temperature (▯ C)	36.1-38.0	0
	35.1-36.0 or 38.1-39.0	1
	>39.0	2
	<35.1	3
Alertness	Alert	0
	Confused or not alert	3
Inspired oxygen	Air	0
	Supplemental oxygen	2
Sex	Female	0
	Male	1
Age (years)	16-49	0
	50-65	2
	66-80	3
	>80	4
Performance status	Unrestricted normal activity	0
	Limited strenuous activity, can do light activity	1
	Limited activity, can self-care	2
	Limited self-care	3
	Bed/chair bound, no self-care	4

Missing data in NEWS2 parameters was handled in the same way as for NEWS2. Performance status was be assumed normal if a clinical frailty scale has not been completed in linked GP records

Supplementary Material 2: Data Sources and linkage

Health and social care data relating to the population in England within the UK National Health Service (NHS) is managed by NHS Digital. We provided patient identifiers to NHS Digital to trace patients in our cohort and supply additional individual level demographic, co-morbidity and outcome data. NHS Digital identified records in their collections belonging to patients in our cohort, and provided data on patient demographics, limited COVID-related general practice (GP) records, emergency department attendances, hospital inpatient admissions, critical care periods, and death registrations from the UK Office of National Statistics.

YAS and NHS Digital removed records where patients indicated that they did not wish their data to be used for research purposes, via the NHS data opt-out service.[15] The study team also excluded patients who had opted out of any part of the PRIEST study and those with inconsistent records (e.g. multiple deaths recorded or death before latest activity). Patient identifiers across all datasets were replaced with a consistent pseudo-identifier to enable the identification and linkage of records belonging to the same patient across all datasets but without revealing any patient's identity.

Supplementary Material 3: Sample Size calculation (Precision of AUROC in cohort 6000 patients with a 5% event rate).

C-statistic	Standard error
0.85	0.014
0.80	0.016
0.75	0.017
0.70	0.017
0.65	0.018

Supplementary Material 4: Performance of triage tools across the whole range of available scores

		Primary: any adverse outcome		Secondary: death	
**Tool**	**Threshold**	**Sensitivity**	**Specificity**	**Sensitivity**	**Specificity**
CRB-65	>0	0.89 (0.88,0.89)	0.54 (0.53,0.54)	0.95 (0.95,0.96)	0.53 (0.53,0.54)
	>1	0.6 (0.59,0.61)	0.83 (0.83,0.84)	0.71 (0.7,0.72)	0.83 (0.83,0.84)
	>2	0.25 (0.24,0.26)	0.96 (0.96,0.96)	0.3 (0.29,0.32)	0.96 (0.96,0.96)
	>3	0.05 (0.04,0.05)	1 (1,1)	0.06 (0.05,0.06)	1 (1,1)
NEWS2	>0	0.99 (0.99,0.99)	0.16 (0.16,0.16)	0.99 (0.98,0.99)	0.15 (0.15,0.16)
	>1	0.96 (0.96,0.96)	0.3 (0.29,0.3)	0.95 (0.95,0.96)	0.28 (0.28,0.29)
	>2	0.93 (0.93,0.94)	0.41 (0.41,0.42)	0.93 (0.93,0.94)	0.4 (0.39,0.4)
	>3	0.89 (0.88,0.9)	0.53 (0.52,0.53)	0.89 (0.88,0.9)	0.51 (0.5,0.51)
	>4	0.84 (0.83,0.84)	0.62 (0.62,0.63)	0.83 (0.82,0.84)	0.6 (0.6,0.61)
	>5	0.77 (0.76,0.77)	0.7 (0.7,0.71)	0.77 (0.76,0.78)	0.69 (0.68,0.69)
	>6	0.65 (0.64,0.66)	0.78 (0.78,0.79)	0.64 (0.63,0.65)	0.76 (0.76,0.77)
	>7	0.51 (0.5,0.52)	0.85 (0.85,0.85)	0.51 (0.5,0.52)	0.83 (0.83,0.84)
	>8	0.39 (0.38,0.4)	0.9 (0.9,0.91)	0.4 (0.39,0.41)	0.89 (0.89,0.9)
	>9	0.27 (0.26,0.28)	0.95 (0.94,0.95)	0.28 (0.27,0.29)	0.94 (0.94,0.94)
	>10	0.18 (0.18,0.19)	0.97 (0.97,0.97)	0.2 (0.19,0.21)	0.97 (0.96,0.97)
	>11	0.12 (0.11,0.13)	0.98 (0.98,0.98)	0.13 (0.12,0.14)	0.98 (0.98,0.98)
	>12	0.06 (0.06,0.07)	0.99 (0.99,0.99)	0.07 (0.06,0.07)	0.99 (0.99,0.99)
	>13	0.04 (0.03,0.04)	1 (1,1)	0.04 (0.04,0.05)	1 (1,1)
	>14	0.02 (0.01,0.02)	1 (1,1)	0.02 (0.01,0.02)	1 (1,1)
	>15	0.01 (0.01,0.01)	1 (1,1)	0.01 (0.01,0.01)	1 (1,1)
	>16	0 (0,0)	1 (1,1)	0 (0,0.01)	1 (1,1)
	>17	0 (0,0)	1 (1,1)	0 (0,0)	1 (1,1)
PMEWS	>0	1 (1,1)	0.08 (0.07,0.08)	1 (1,1)	0.07 (0.07,0.08)
	>1	0.99 (0.99,0.99)	0.2 (0.2,0.21)	0.99 (0.99,1)	0.2 (0.19,0.2)
	>2	0.98 (0.97,0.98)	0.34 (0.33,0.34)	0.98 (0.98,0.98)	0.33 (0.32,0.33)
	>3	0.94 (0.94,0.95)	0.47 (0.46,0.47)	0.95 (0.94,0.95)	0.45 (0.45,0.46)
	>4	0.89 (0.88,0.89)	0.58 (0.58,0.59)	0.89 (0.88,0.9)	0.56 (0.56,0.57)
	>5	0.8 (0.8,0.81)	0.68 (0.67,0.68)	0.82 (0.81,0.83)	0.66 (0.66,0.66)
	>6	0.69 (0.69,0.7)	0.77 (0.77,0.78)	0.71 (0.7,0.72)	0.76 (0.75,0.76)
	>7	0.55 (0.54,0.56)	0.85 (0.84,0.85)	0.57 (0.56,0.59)	0.83 (0.83,0.84)
	>8	0.41 (0.4,0.42)	0.9 (0.9,0.9)	0.43 (0.42,0.44)	0.89 (0.89,0.89)
	>9	0.29 (0.29,0.3)	0.94 (0.94,0.94)	0.31 (0.3,0.32)	0.94 (0.93,0.94)
	>10	0.19 (0.18,0.19)	0.97 (0.97,0.97)	0.21 (0.2,0.22)	0.97 (0.97,0.97)
	>11	0.11 (0.11,0.12)	0.99 (0.99,0.99)	0.13 (0.12,0.14)	0.99 (0.99,0.99)
	>12	0.07 (0.07,0.08)	1 (1,1)	0.08 (0.08,0.09)	1 (0.99,1)
	>13	0.04 (0.03,0.04)	1 (1,1)	0.04 (0.04,0.05)	1 (1,1)
	>14	0.01 (0.01,0.02)	1 (1,1)	0.02 (0.01,0.02)	1 (1,1)
	>15	0.01 (0.01,0.01)	1 (1,1)	0.01 (0.01,0.01)	1 (1,1)

		Primary: any adverse outcome		Secondary: death	
**Tool**	**Threshold**	**Sensitivity**	**Specificity**	**Sensitivity**	**Specificity**
	>16	0 (0,0)	1 (1,1)	0 (0,0)	1 (1,1)
PRIEST	>0	1 (1,1)	0.05 (0.05,0.05)	1 (1,1)	0.05 (0.05,0.05)
	>1	1 (1,1)	0.12 (0.12,0.13)	1 (1,1)	0.12 (0.12,0.12)
	>2	1 (0.99,1)	0.21 (0.21,0.22)	1 (1,1)	0.21 (0.2,0.21)
	>3	0.99 (0.99,0.99)	0.31 (0.3,0.31)	0.99 (0.99,1)	0.29 (0.29,0.3)
	>4	0.97 (0.97,0.97)	0.41 (0.4,0.41)	0.98 (0.97,0.98)	0.39 (0.39,0.4)
	>5	0.95 (0.95,0.95)	0.49 (0.49,0.5)	0.96 (0.95,0.96)	0.48 (0.47,0.48)
	>6	0.92 (0.92,0.93)	0.57 (0.56,0.57)	0.94 (0.93,0.95)	0.55 (0.55,0.55)
	>7	0.89 (0.88,0.89)	0.64 (0.63,0.64)	0.91 (0.9,0.92)	0.62 (0.62,0.62)
	>8	0.83 (0.82,0.83)	0.7 (0.7,0.71)	0.86 (0.85,0.87)	0.69 (0.68,0.69)
	>9	0.75 (0.74,0.76)	0.76 (0.76,0.76)	0.8 (0.79,0.81)	0.75 (0.74,0.75)
	>10	0.65 (0.64,0.66)	0.81 (0.81,0.82)	0.7 (0.69,0.71)	0.8 (0.8,0.81)
	>11	0.53 (0.52,0.54)	0.86 (0.86,0.87)	0.59 (0.58,0.6)	0.86 (0.85,0.86)
	>12	0.44 (0.43,0.44)	0.9 (0.9,0.9)	0.5 (0.49,0.51)	0.9 (0.9,0.9)
	>13	0.35 (0.34,0.36)	0.93 (0.93,0.94)	0.41 (0.4,0.42)	0.93 (0.93,0.93)
	>14	0.28 (0.27,0.28)	0.95 (0.95,0.96)	0.33 (0.32,0.34)	0.95 (0.95,0.95)
	>15	0.21 (0.2,0.22)	0.97 (0.97,0.97)	0.25 (0.24,0.26)	0.97 (0.97,0.97)
	>16	0.14 (0.14,0.15)	0.98 (0.98,0.98)	0.17 (0.17,0.18)	0.98 (0.98,0.98)
	>17	0.11 (0.1,0.11)	0.99 (0.99,0.99)	0.13 (0.12,0.14)	0.99 (0.99,0.99)
	>18	0.07 (0.06,0.07)	0.99 (0.99,0.99)	0.08 (0.08,0.09)	0.99 (0.99,0.99)
	>19	0.04 (0.04,0.05)	1 (1,1)	0.05 (0.05,0.06)	1 (1,1)
	>20	0.03 (0.02,0.03)	1 (1,1)	0.03 (0.03,0.04)	1 (1,1)
	>21	0.02 (0.01,0.02)	1 (1,1)	0.02 (0.02,0.02)	1 (1,1)
	>22	0.01 (0.01,0.01)	1 (1,1)	0.01 (0.01,0.01)	1 (1,1)
	>23	0 (0,0.01)	1 (1,1)	0 (0,0.01)	1 (1,1)
	>24	0 (0,0)	1 (1,1)	0 (0,0)	1 (1,1)
	>25	0 (0,0)	1 (1,1)	0 (0,0)	1 (1,1)
WHO	>0	0.98 (0.97,0.98)	0.31 (0.3,0.31)	0.99 (0.99,1)	0.3 (0.3,0.3)

Supplementary 5: Flexible calibration curves using locally weighted scatterplot smoothing (LOESS) with 95% CIs

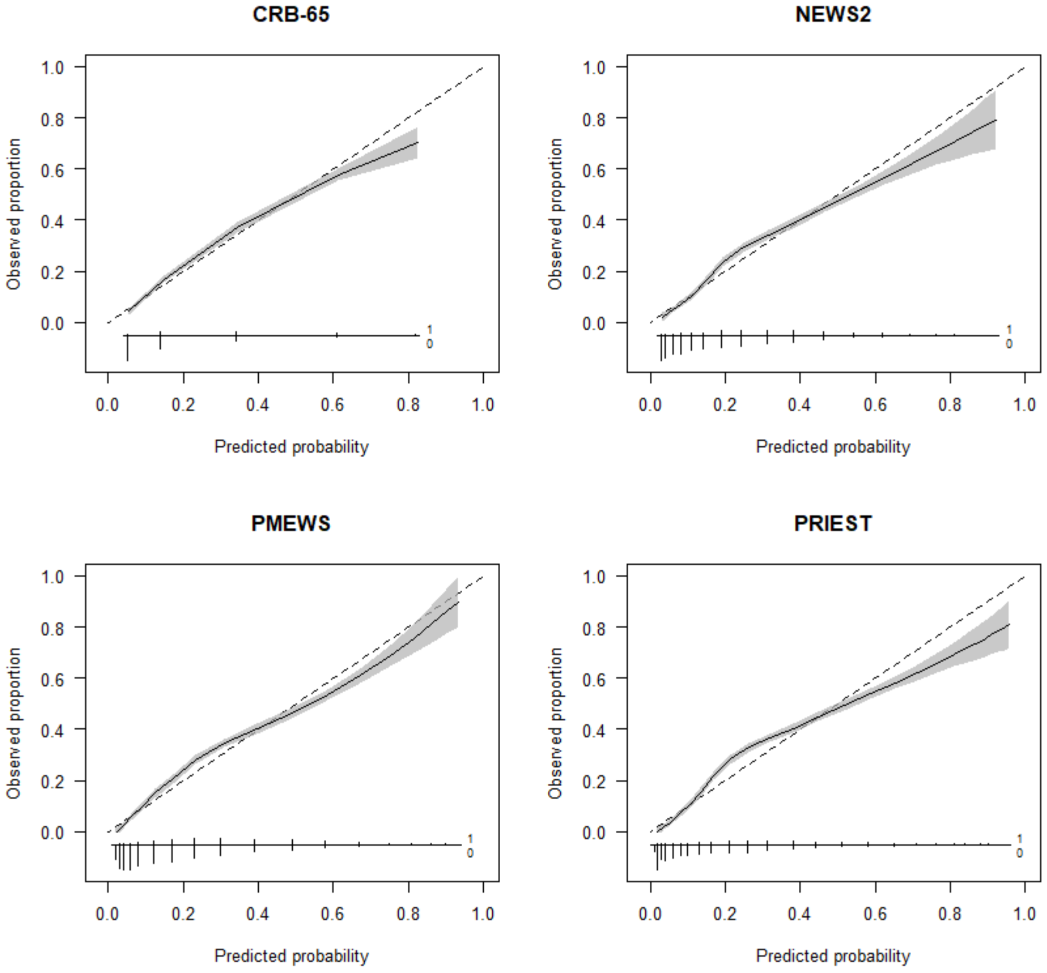

Supplementary Material 6: Triage tool diagnostic accuracy statistics (95% CI) for predicting inpatient admission

Tool	N*	N (%) admitted**	C-statistic	Threshold	N (%) with score	Sensitivity	Specificity	PPV	NPV
CRB-65	7469	3890 (52%)	0.73 (0.72, 0.74)	>0	4010 (54%)	0.75 (0.74, 0.75)	0.69 (0.68, 0.69)	0.72 (0.71, 0.72)	0.71 (0.70, 0.72)
NEWS2	7433	3875 (52%)	0.81 (0.80, 0.82)	>1	5574 (75%)	0.92 (0.91, 0.92)	0.43 (0.43, 0.44)	0.64 (0.63, 0.64)	0.83 (0.82, 0.83)
PMEWS	7460	3890 (52%)	0.83 (0.82, 0.84)	>2	5352 (72%)	0.92 (0.92, 0.92)	0.50 (0.50, 0.51)	0.67 (0.66, 0.67)	0.85 (0.85, 0.86)
PRIEST	7471	3890 (52%)	0.83 (0.82, 0.84)	>4	4932 (66%)	0.90 (0.89, 0.9)	0.60 (0.59, 0.60)	0.71 (0.70, 0.71)	0.84 (0.84, 0.85)
WHO	7471	3890 (52%)	0.67 (0.66, 0.68)	>0	5539 (74%)	0.90 (0.90, 0.90)	0.43 (0.43, 0.44)	0.63 (0.63, 0.64)	0.80 (0.80, 0.81)

*Patients with 3 or more missing triage tool parameters were excluded from analysis when estimating performance

**Numbers rounded to nearest 5
